# DGKα and ζ Deficiency Causes Regulatory T-Cell Dysregulation, Destabilization, and Conversion to Pathogenic T-Follicular Helper Cells to Trigger IgG1-Predominant Autoimmunity

**DOI:** 10.1101/2024.11.26.625360

**Published:** 2024-12-01

**Authors:** Lei Li, Hongxiang Huang, Hongxia Wang, Yun Pan, Huishan Tao, Shimeng Zhang, Peer W.F. Karmaus, Michael B. Fessler, John W. Sleasman, Xiao-Ping Zhong

## Abstract

Regulatory T cells (Tregs) actively engage in immune suppression to prevent autoimmune diseases but also inhibit anti-tumor immunity. Although Tregs express a TCR repertoire with relatively high affinities to self, they are normally quite stable and their inflammatory programs are intrinsically suppressed. We report here that diacylglycerol (DAG) kinases (DGK) ( and ( are crucial for homeostasis, suppression of proinflammatory programs, and stability of Tregs and for enforcing their dependence on CD28 costimulatory signal. Treg-specific deficiency of both DGK( and ( derails signaling, metabolic, and transcriptional programs in Tregs to cause dysregulated phenotypic and functional properties and to unleash conversion to pathogenic exTregs, especially exTreg-T follicular helper (Tfh) 2 cells, leading to uncontrolled effector T cell differentiation, deregulated germinal center (GC) B-cell responses and IgG1/IgE predominant antibodies/autoantibodies, and multiorgan autoimmune diseases. Our data not only illustrate the crucial roles of DGKs in Tregs to maintain self-tolerance but also unveil a Treg-to-self-reactive-pathogenic-exTreg-Tfh-cell program that is suppressed by DGKs and that could exert broad pathogenic roles in autoimmune diseases if unchecked.

## Full Text Availability

The license terms selected by the author(s) for this preprint version do not permit archiving in PMC. The full text is available from the preprint server.

