# Supplementary material for: DGKα and ζ Deficiency Causes Regulatory T-Cell Dysregulation, Destabilization, and Conversion to Pathogenic T-Follicular Helper Cells to Trigger IgG1-Predominant Autoimmunity
Source: bioRxiv. 2025 May 19:2024.11.26.625360. Originally published 2024 Dec 1. Preprint. [Version 2] doi: 10.1101/2024.11.26.625360 (PMC11623591; doi:10.1101/2024.11.26.625360)
Supplement: Supplement 2 [file media-2.pdf]

Supplemental Figure S2

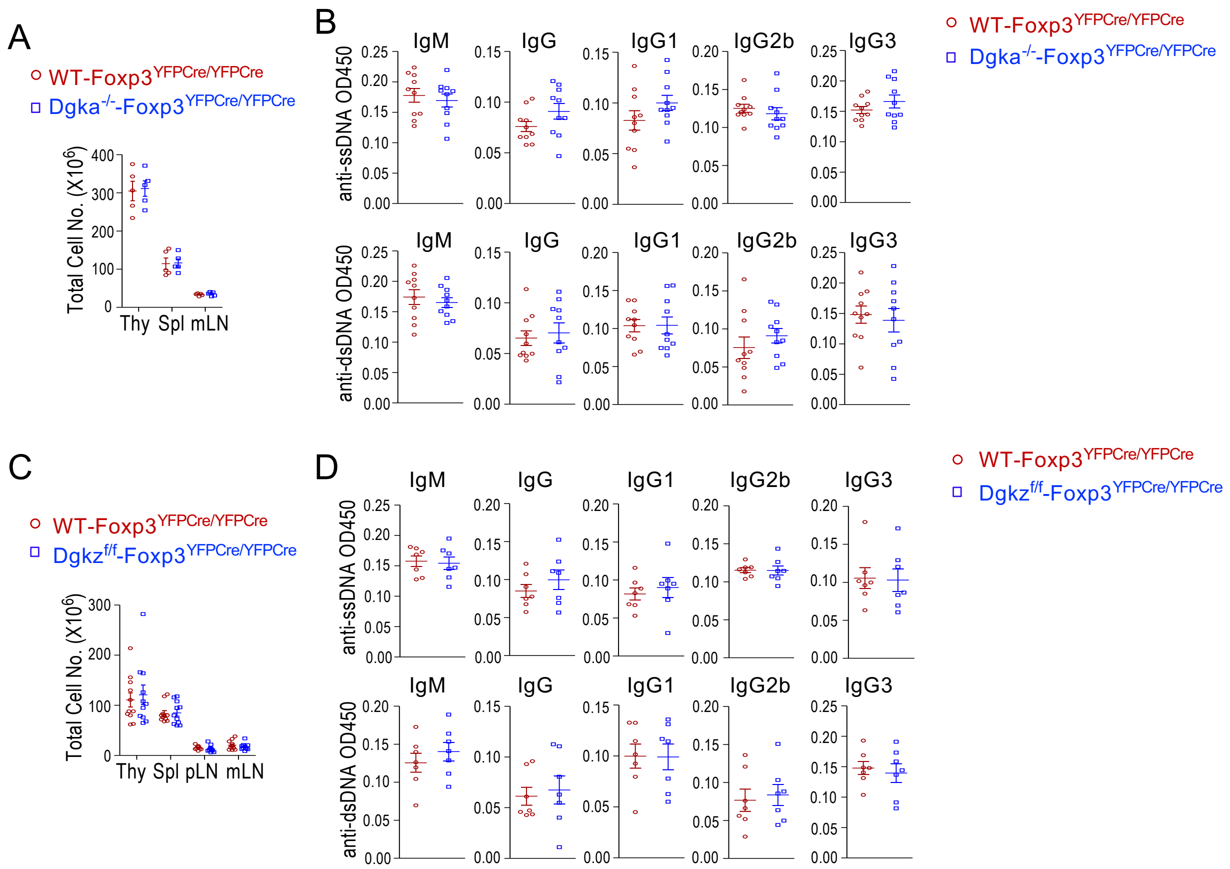

Supplemental Figure S2. Analyses of *Dgka*$^{-/-}$-*Foxp3*$^{YFPCre/YFPCre}$ mice and *Dgka*$^{+/+}$*z*$^{f/f}$-*Foxp3*$^{YFPCre/YFPCre}$ mice. A–B. *Dgka*$^{-/-}$-*Foxp3*$^{YFPCre/YFPCre}$ mice and WT-*Foxp3*$^{YFPCre/YFPCre}$ mice. **A.** Total cellularity. **B.** Serum autoantibodies. **C–D.** *Dgka*$^{+/+}$*z*$^{f/f}$-*Foxp3*$^{YFPCre/YFPCre}$ mice and WT-*Foxp3*$^{YFPCre/YFPCre}$ mice. **C.** Total cellularity. **D.** Serum autoantibodies. Each circle or square represents one mouse of the indicated genotypes. Data shown are presentative of or pooled from at least five experiments.
