# Supplementary material for: DGKα and ζ Deficiency Causes Regulatory T-Cell Dysregulation, Destabilization, and Conversion to Pathogenic T-Follicular Helper Cells to Trigger IgG1-Predominant Autoimmunity
Source: bioRxiv. 2025 May 19:2024.11.26.625360. Originally published 2024 Dec 1. Preprint. [Version 2] doi: 10.1101/2024.11.26.625360 (PMC11623591; doi:10.1101/2024.11.26.625360)
Supplement: Supplement 3 [file media-3.pdf]

Supplemental Figure S3

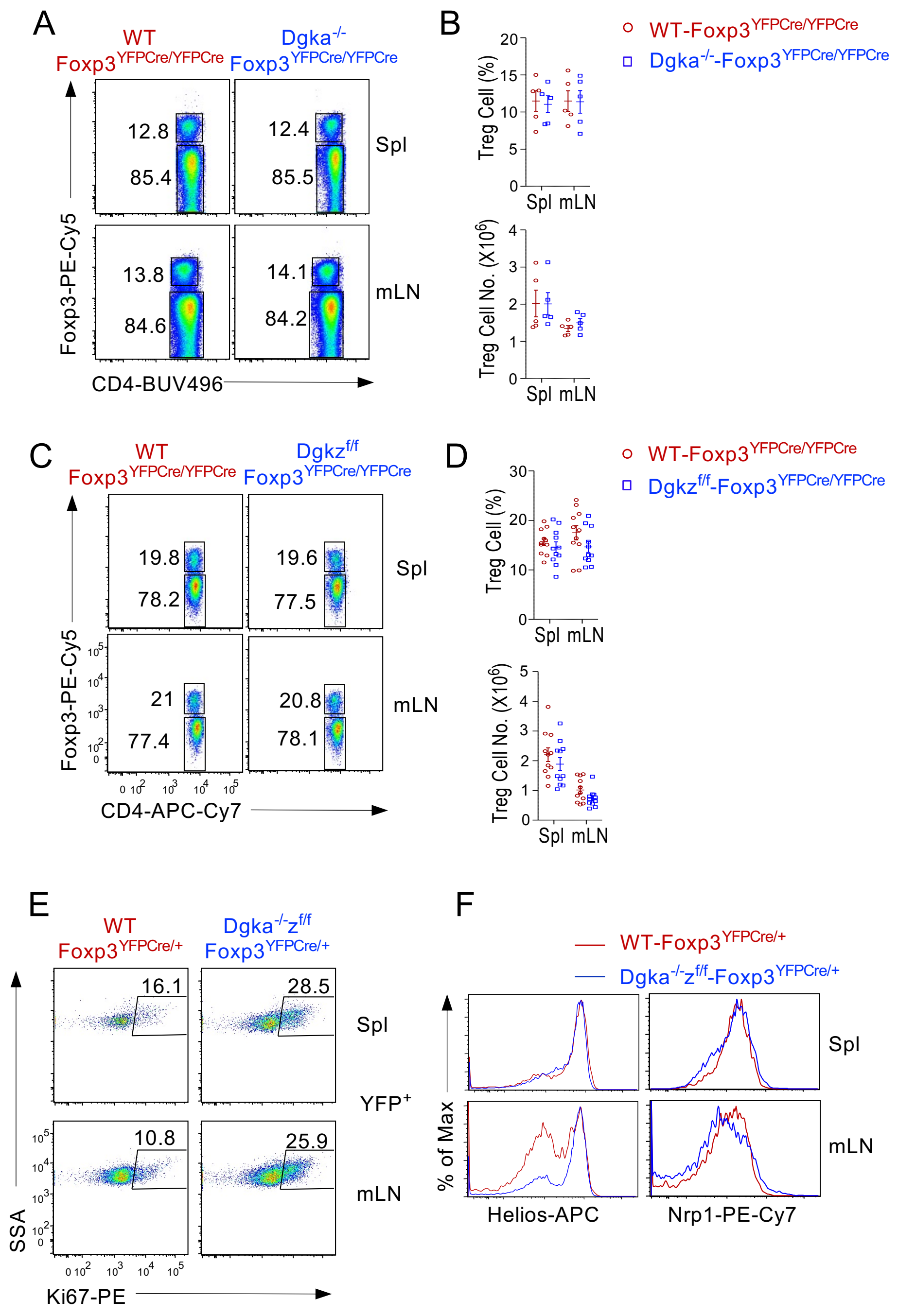

Supplemental Figure S3. Analyses of Tregs in DGKα or DGKζ single knockout mice. A, B. Tregs in Dgka$^{-/-}$ mice. **C, D.** Tregs of *Dgka$^{+/+}$z$^{f/f}$-Foxp3$^{YFPCre/YFPCre}$* mice. **E, F.** Analysis of female *Dgka$^{-/-}$z$^{f/f}$-Foxp3$^{YFPCre/+}$* and WT-*Foxp3$^{YFPCre/+}$* mice. **E.** Ki67 staining in YFP^{+} Tregs. **F.** Overlaid histograms showing Helios and Nrp1 expression in YFP^{+} Tregs. Each circle or square represents one mouse of the indicated genotypes. Data shown are presentative of or pooled from at least five experiments.
