# Supplementary material for: DGKα and ζ Deficiency Causes Regulatory T-Cell Dysregulation, Destabilization, and Conversion to Pathogenic T-Follicular Helper Cells to Trigger IgG1-Predominant Autoimmunity
Source: bioRxiv. 2025 May 19:2024.11.26.625360. Originally published 2024 Dec 1. Preprint. [Version 2] doi: 10.1101/2024.11.26.625360 (PMC11623591; doi:10.1101/2024.11.26.625360)
Supplement: Supplement 5 [file media-5.pdf]

Supplemental Figure S5

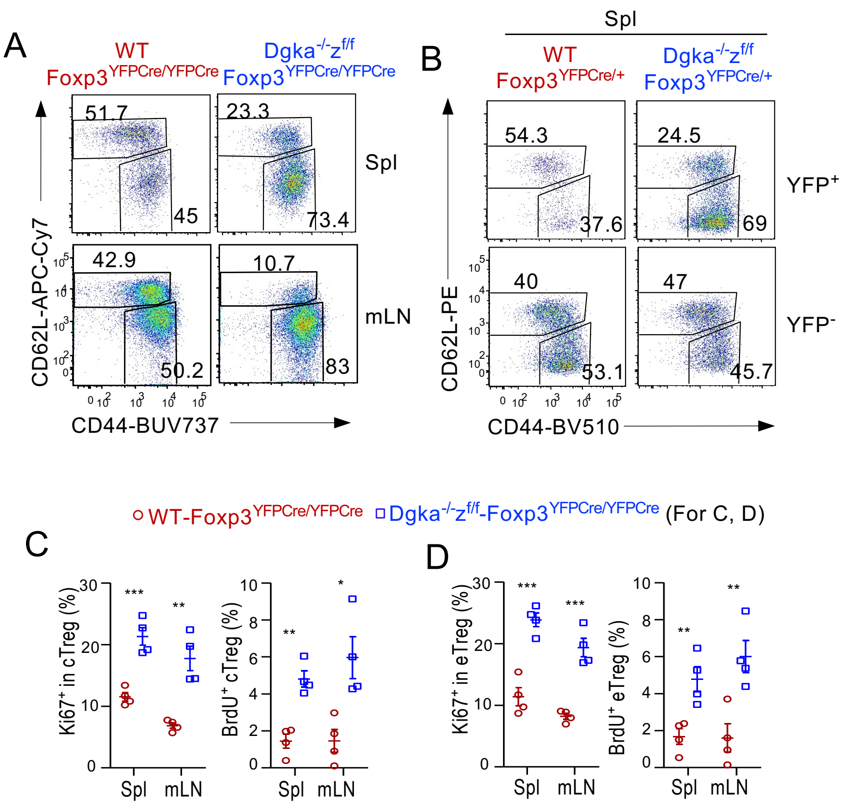

Supplemental Figure S5. A. CD44 and CD62L expression in CD4⁺Foxp3⁺ Tregs from *Dgka⁻/⁻z^f/f-Foxp3^YFPCre/YFPCre* and WT-*Foxp3^YFPCre/YFPCre* control mice. **B.** CD44 and CD62L expression in YFP⁺ and YFP⁻ CD4⁺Foxp3⁺ Tregs from female *Dgka⁻/⁻z^f/f-Foxp3^YFPCre/+* and WT- *Foxp3^YFPCre/+* control mice. **C, D.** Scatter plots show mean ± SEM of percentages of Ki67⁺ or BrdU⁺ cells within cTregs (C) and eTregs (D).
