# Supplementary material for: DGKα and ζ Deficiency Causes Regulatory T-Cell Dysregulation, Destabilization, and Conversion to Pathogenic T-Follicular Helper Cells to Trigger IgG1-Predominant Autoimmunity
Source: bioRxiv. 2025 May 19:2024.11.26.625360. Originally published 2024 Dec 1. Preprint. [Version 2] doi: 10.1101/2024.11.26.625360 (PMC11623591; doi:10.1101/2024.11.26.625360)
Supplement: Supplement 6 [file media-6.pdf]

Supplemental Figure S6

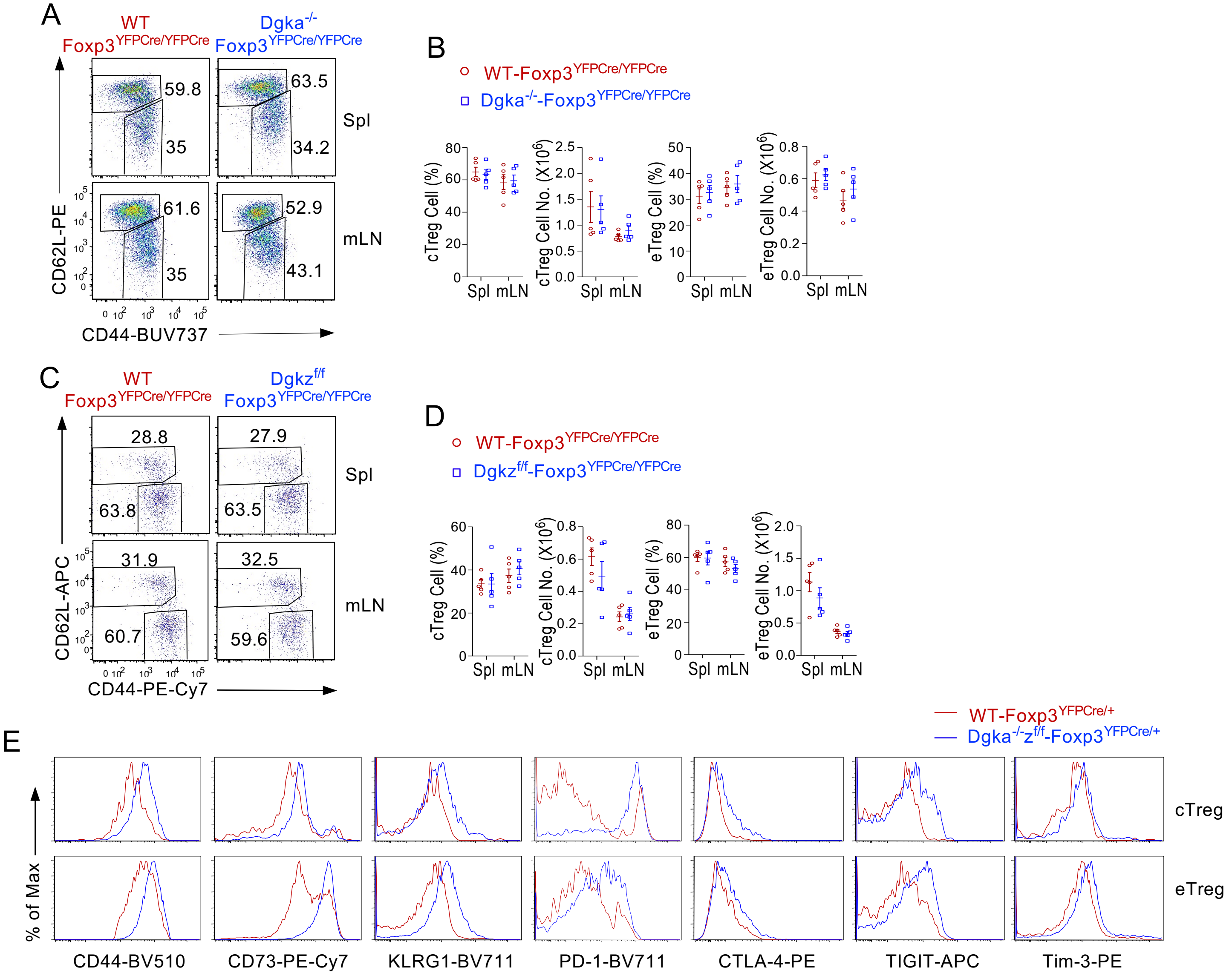

Supplemental Figure S6. Treg properties in the absence of DGKα, ζ, or both. A, B. Analyses of *Dgka*$^{-/-}$*z*$^{+/+}$-*Foxp3*$^{YFPCre/YFPCre}$ and WT-*Foxp3*$^{YFPCre/YFPCre}$ control mice. **A.** CD44 and CD62L expression in CD4^{+}Foxp3^{+} Tregs. **B.** Scatter plots show mean ± SEM of cTreg and eTreg percentages and numbers. **C, D.** Analyses of *Dgka*$^{+/+}$*z*$^{f/f}$-*Foxp3*$^{YFPCre/YFPCre}$ and WT-*Foxp3*$^{YFPCre/YFPCre}$ control mice. **C.** CD44 and CD62L expression in CD4^{+}Foxp3^{+} Tregs. **D.** Scatter plots show mean ± SEM of cTreg and eTreg percentages and numbers. **E.** Overlaid histograms comparing expression of indicated molecules in YFP^{+} Tregs from female *Dgka*$^{-/-}$*z*$^{f/f}$-*Foxp3*$^{YFPCre/+}$ and WT-*Foxp3*$^{YFPCre/+}$ control mice. Data shown are presentative of or pooled from at least five experiments.
