# Supplementary material for: DGKα and ζ Deficiency Causes Regulatory T-Cell Dysregulation, Destabilization, and Conversion to Pathogenic T-Follicular Helper Cells to Trigger IgG1-Predominant Autoimmunity
Source: bioRxiv. 2025 May 19:2024.11.26.625360. Originally published 2024 Dec 1. Preprint. [Version 2] doi: 10.1101/2024.11.26.625360 (PMC11623591; doi:10.1101/2024.11.26.625360)
Supplement: Supplement 7 [file media-7.pdf]

Supplemental Figure S7

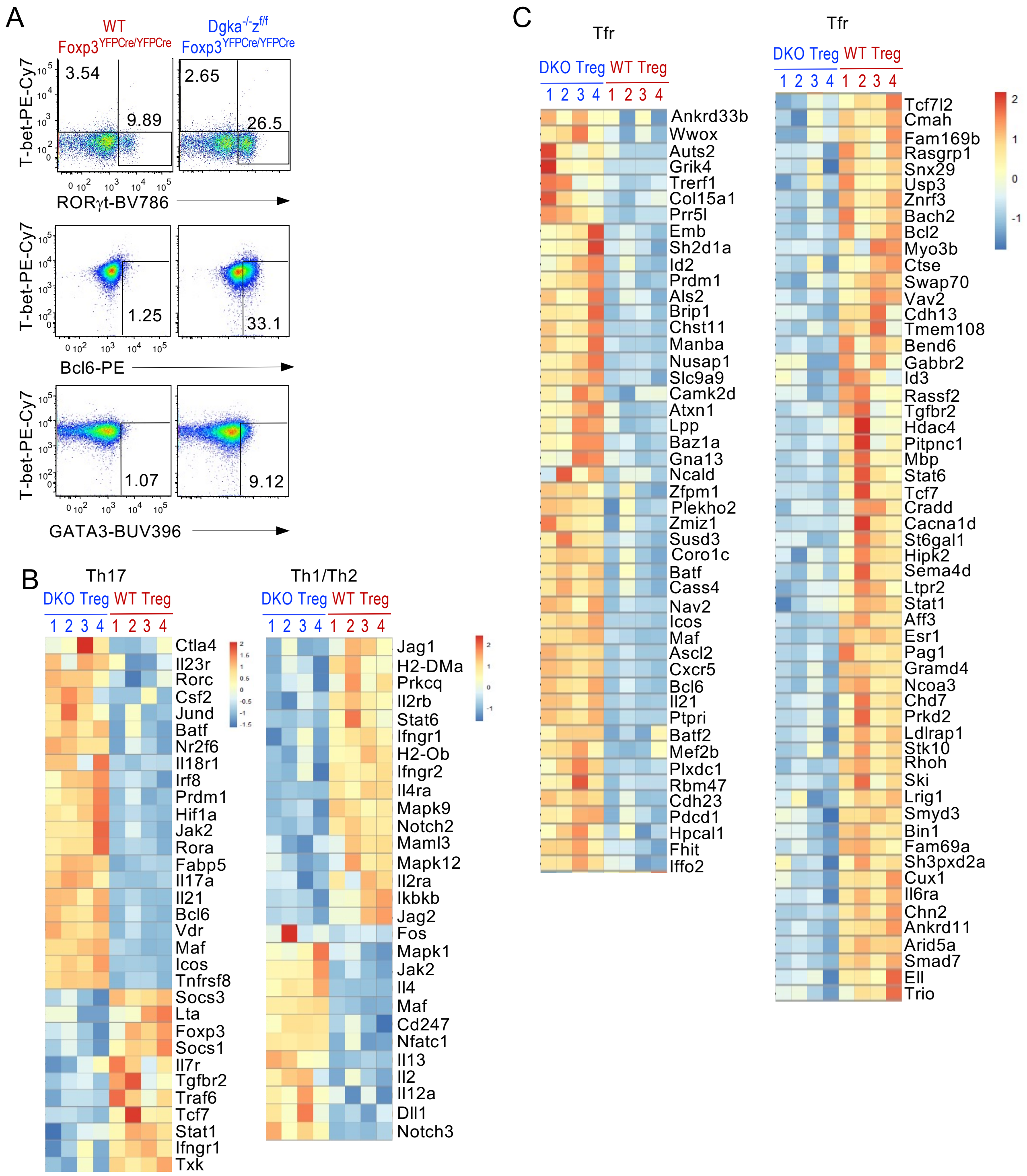

Supplemental Figure S7. Analysis of effector lineages and gain-of-proinflammatory properties of αζDKO Tregs. A. Intracellular staining of TF in mLN Tregs in WT-*Foxp3*$^{YFPCre/YFPCre}$ and *Dgka*$^{-/-}$*z*$^{f/f}$-*Foxp3*$^{YFPCre/YFPCre}$ mice. **B, C.** Heatmaps showing altered Th1, Th2, and Th17 cell (B) and Tfr cell (C) associated genes with statistically significant differences between WT and αζDKO Tregs ($p<0.05$).
