# Supplementary material for: DGKα and ζ Deficiency Causes Regulatory T-Cell Dysregulation, Destabilization, and Conversion to Pathogenic T-Follicular Helper Cells to Trigger IgG1-Predominant Autoimmunity
Source: bioRxiv. 2025 May 19:2024.11.26.625360. Originally published 2024 Dec 1. Preprint. [Version 2] doi: 10.1101/2024.11.26.625360 (PMC11623591; doi:10.1101/2024.11.26.625360)
Supplement: Supplement 8 [file media-8.pdf]

Supplemental Figure S8

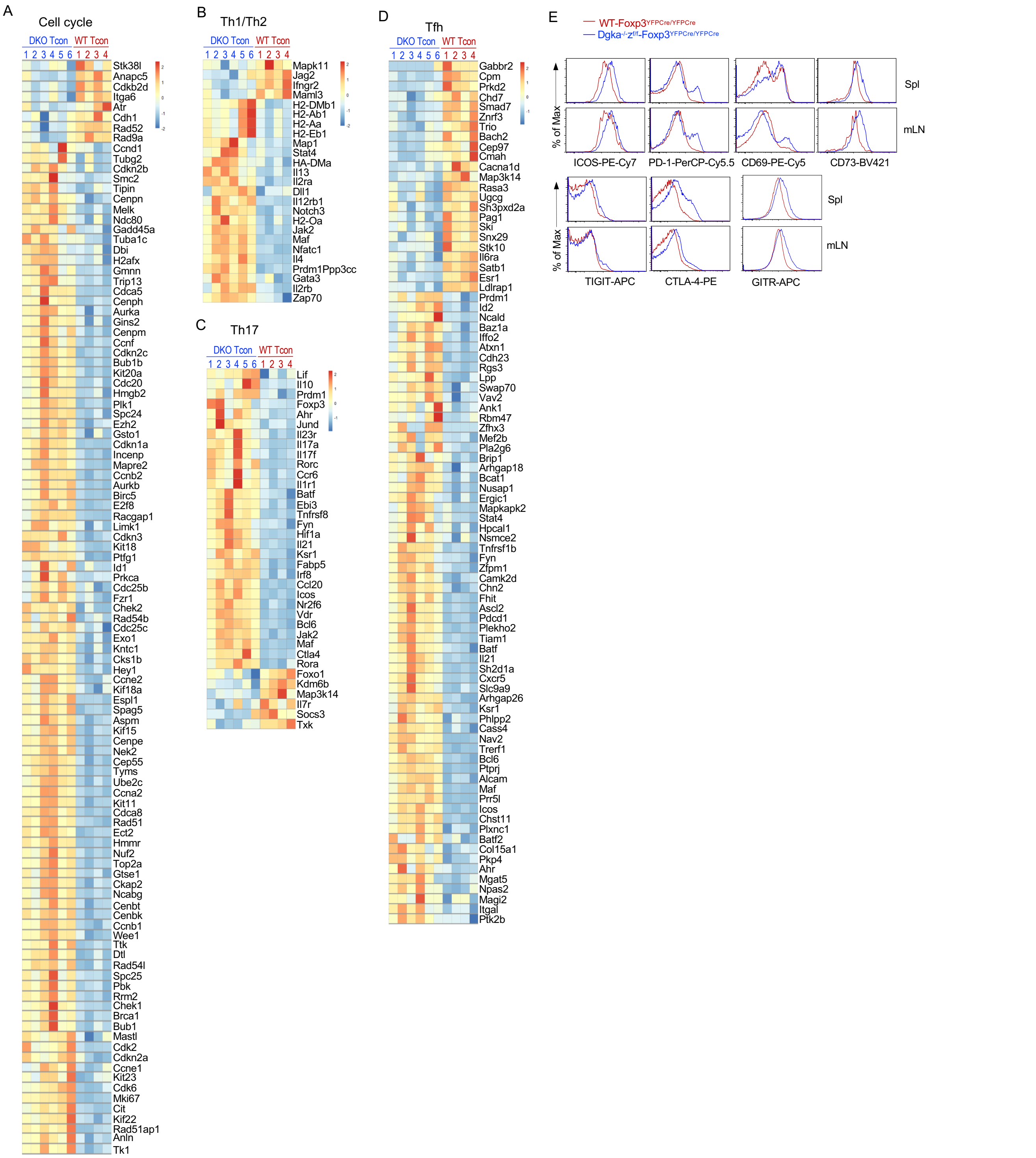

Supplemental Figure S8. Altered cell cycle and Th associated pathways in CD4⁺Foxp3⁻ Tcons from Treg-αζDKO mice. A. Heatmap shows DE cell cycle associated genes. **B.** Heatmap shows DE Th1/2 associated genes. **C.** Heatmap shows DE Th17 associated genes. **D.** Heatmap shows DE Tfh/Tfr associated genes. **E.** Alerted expression of surface molecules in Tcons from *Dgka*$^{-/-}$-*z*$^{f/f}$-*Foxp3*$^{YFPCre/YFPCre}$ and WT-*Foxp3*$^{YFPCre/YFPCre}$ mice.
