# Supplementary material for: DGKα and ζ Deficiency Causes Regulatory T-Cell Dysregulation, Destabilization, and Conversion to Pathogenic T-Follicular Helper Cells to Trigger IgG1-Predominant Autoimmunity
Source: bioRxiv. 2025 May 19:2024.11.26.625360. Originally published 2024 Dec 1. Preprint. [Version 2] doi: 10.1101/2024.11.26.625360 (PMC11623591; doi:10.1101/2024.11.26.625360)
Supplement: Supplement 10 [file media-10.pdf]

# Supplemental Figure S10

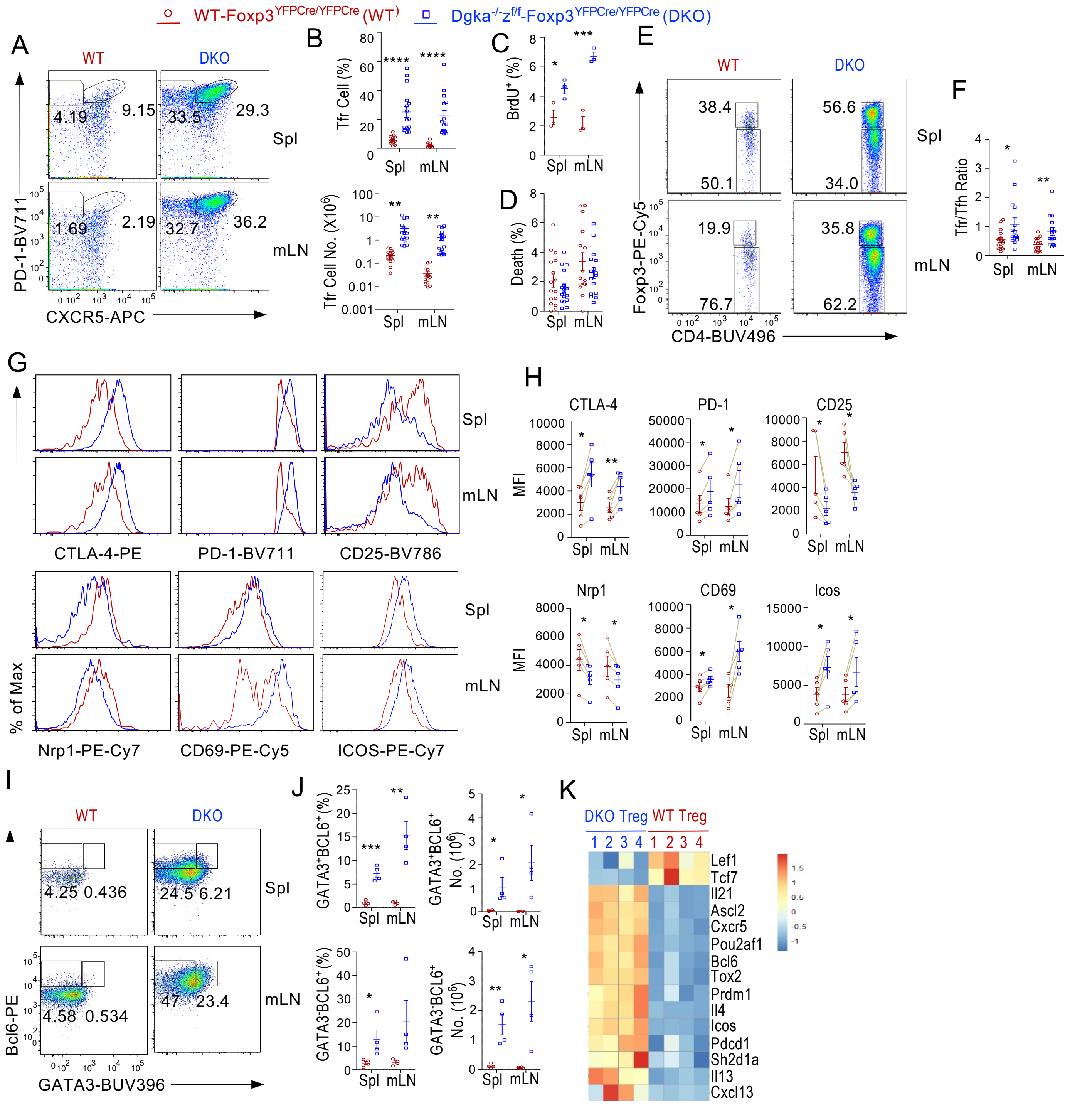


**Supplemental Figure S10. Dysregulation of Tfr cells in *Dgka$^{-/-}$z$^{f/f}$-Foxp3$^{YFPCre/YFPCre}$* mice.** Splenocytes and LN cells from *Dgka$^{-/-}$z$^{f/f}$-Foxp3$^{YFPCre/YFPCre}$* and WT-*Foxp3$^{YFPCre/YFPCre}$* mice were analyzed. **A.** CXCR5 and PD-1 expression in CD4$^+$Foxp3$^+$ Tregs. **B.** Mean ± SEM of Tfr percentages and numbers. **C.** BrdU incorporation in Tfr cells. **D.** Death rates of Tfr cells. **E.** Foxp3 and CD4 expression in CXCR5$^+$PD-1$^+$ CD4$^+$ T cells. **F.** Mean ± SEM of Tfr/Tcon ratios. **G.** Representative histograms show expression of indicated molecules in Tfr cells. **H.** Scatter plots represent mean ± SEM of MFI of the indicated molecules in Tfr cells. **I.** Bcl6 and GATA3 expression in CD4$^+$Foxp3$^+$ Tregs. **J.** Mean ± SEM of percentages and numbers of GATA3$^+$Bcl6$^+$ and GATA3$^-$Bcl6$^+$ cells in Tregs. **K.** Heatmap showing expression of Tfr-/Tfh-cell-related genes in WT and DKO Tregs. Data shown are representative of or pooled from 3–16 experiments. *, $p < 0.05$; **, $p < 0.01$; ***, $p < 0.001$; ****, $p < 0.0001$ determined by two-tail unpaired Student *t* test (B – D, F and J) or pairwise Student t-test for H.
