# Supplementary material for: DGKα and ζ Deficiency Causes Regulatory T-Cell Dysregulation, Destabilization, and Conversion to Pathogenic T-Follicular Helper Cells to Trigger IgG1-Predominant Autoimmunity
Source: bioRxiv. 2025 May 19:2024.11.26.625360. Originally published 2024 Dec 1. Preprint. [Version 2] doi: 10.1101/2024.11.26.625360 (PMC11623591; doi:10.1101/2024.11.26.625360)
Supplement: Supplement 11 [file media-11.pdf]

Supplemental Figure S11

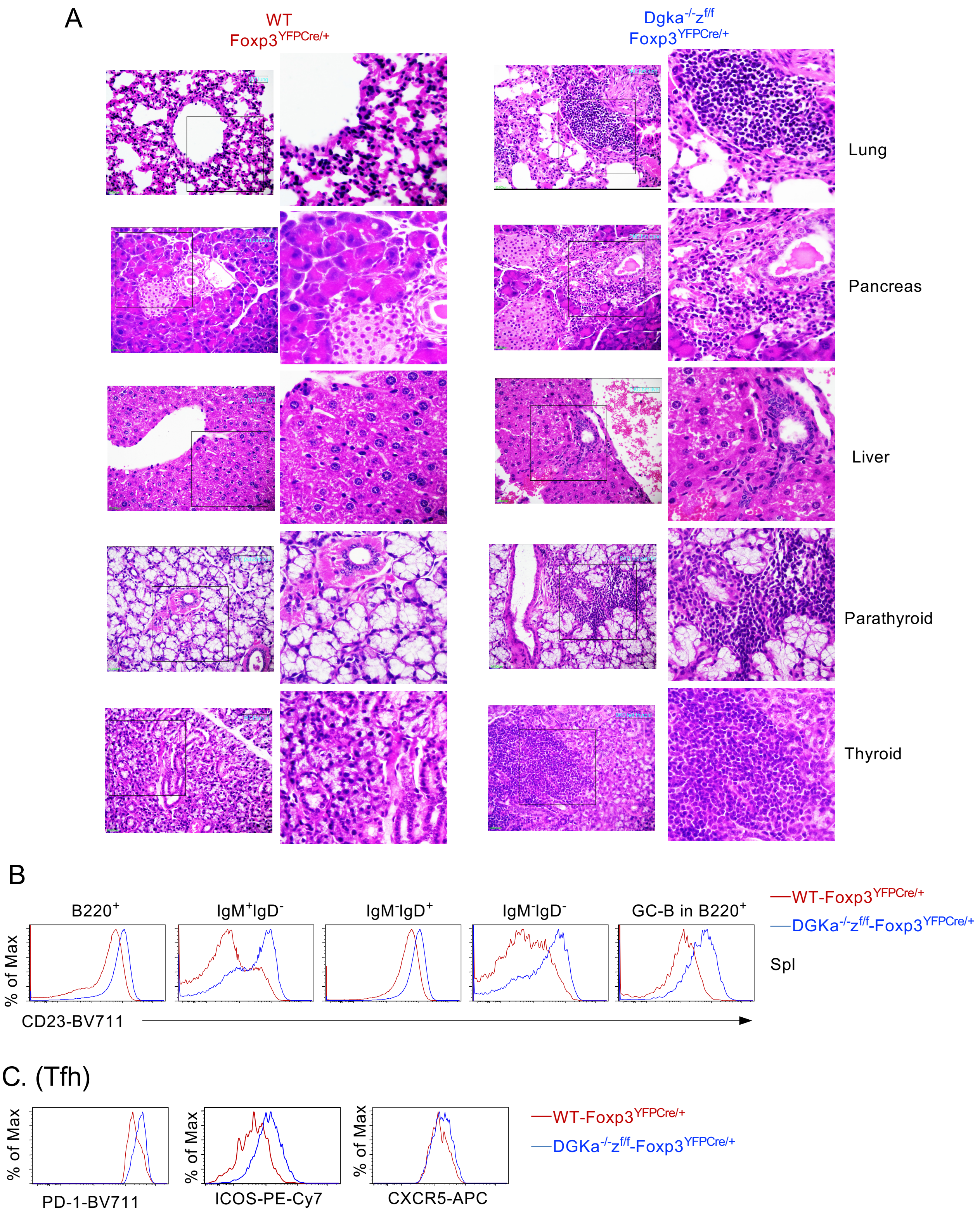

Supplemental Figure S11. Development of autoimmune diseases in female *Dgka$^{-/-}$z$^{f/f}$-Foxp3$^{YFPCre/+}$* mice. Three – nine months old female *Dgka$^{-/-}$z$^{f/f}$-Foxp3$^{YFPCre/+}$* (DKO-Cre^{het}) and WT-*Foxp3$^{YFPCre/+}$* (WT-Cre^{het}) mice were analyzed. **A.** Representative H&E staining of thin sections of the indicated organs. **B.** CD23 levels in the indicated B cell populations. **C.** PD-1, ICOS, and CXCR5 levels in Tfh cells.
