# Supplementary material for: DGKα and ζ Deficiency Causes Regulatory T-Cell Dysregulation, Destabilization, and Conversion to Pathogenic T-Follicular Helper Cells to Trigger IgG1-Predominant Autoimmunity
Source: bioRxiv. 2025 May 19:2024.11.26.625360. Originally published 2024 Dec 1. Preprint. [Version 2] doi: 10.1101/2024.11.26.625360 (PMC11623591; doi:10.1101/2024.11.26.625360)
Supplement: Supplement 12 [file media-12.pdf]

Supplemental Figure S12

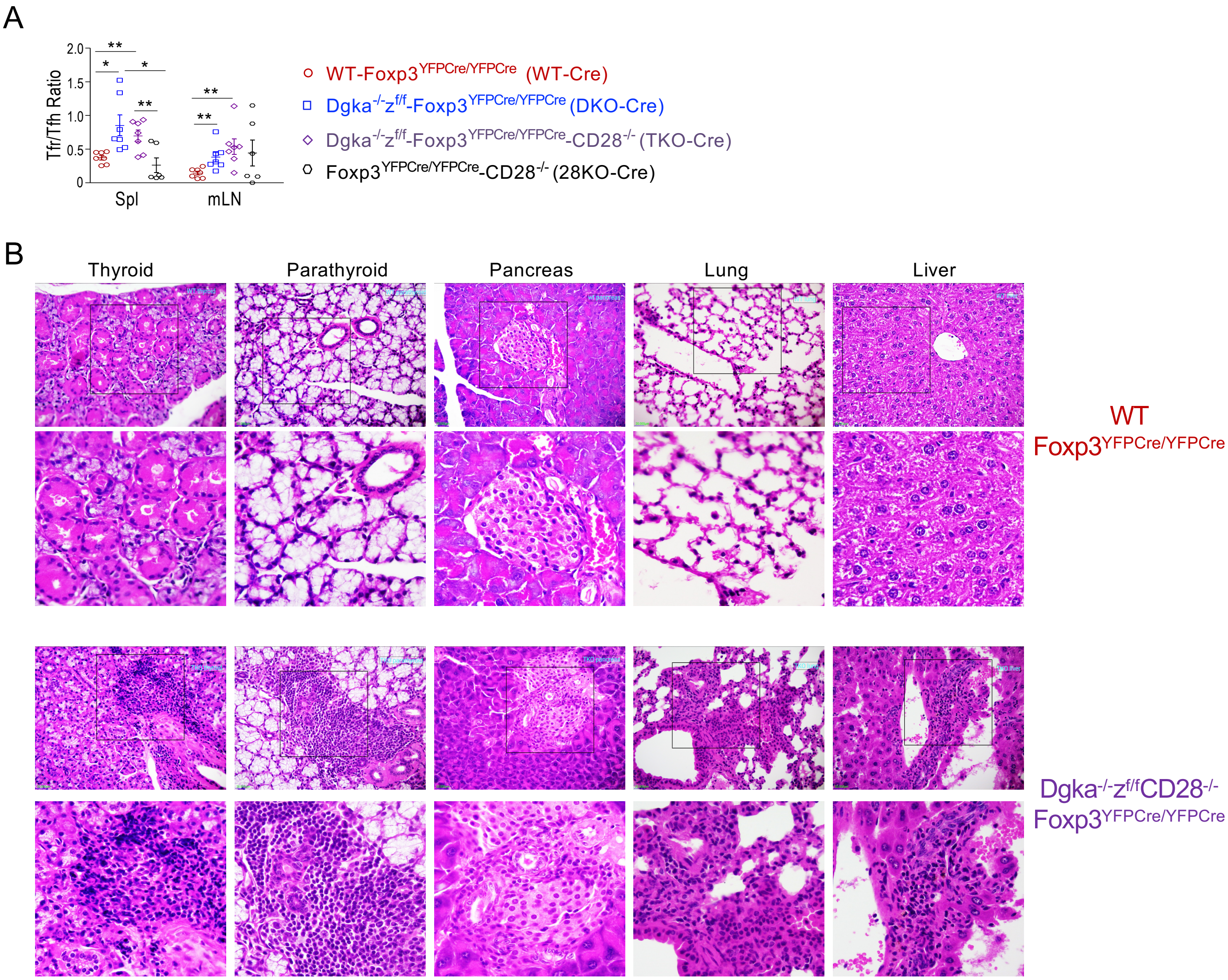

Supplemental Figure S12. Altered Tfr/Tfh cell ratios and development of autoimmune diseases in *Dgka$^{-/-}$z$^{f/f}$-Foxp3$^{YFPCre/YFPCre}$-CD28$^{-/-}$* mice. A. Tfr/Tfh cell ratios in the indicated mice. **B.** Representative H&E staining of tissue thin sections in the indicated mice are shown. Data shown are pooled from 5–10 experiments. *, $p < 0.05$; **, $p < 0.01$ determined by two-tail unpaired Student *t* test.
