# Supplementary material for: DGKα and ζ Deficiency Causes Regulatory T-Cell Dysregulation, Destabilization, and Conversion to Pathogenic T-Follicular Helper Cells to Trigger IgG1-Predominant Autoimmunity
Source: bioRxiv. 2025 May 19:2024.11.26.625360. Originally published 2024 Dec 1. Preprint. [Version 2] doi: 10.1101/2024.11.26.625360 (PMC11623591; doi:10.1101/2024.11.26.625360)
Supplement: Supplement 13 [file media-13.pdf]

Supplemental Figure S13

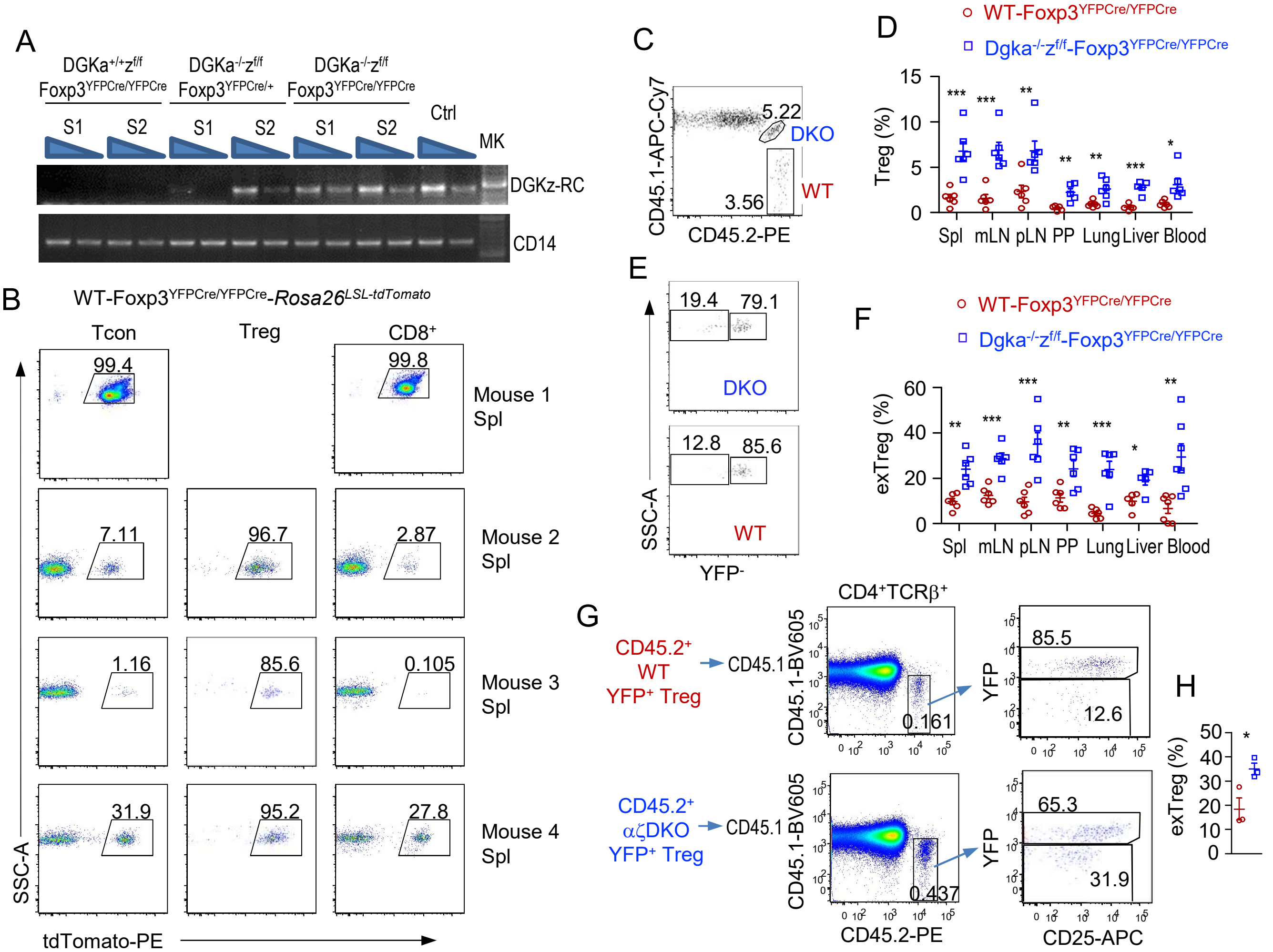

Supplemental Figure S13. Enhanced αζDKO Treg conversion to exTregs/exTreg-Tfh cells. A. Detection of Cre mediated recombination in CD4^{+}Foxp3YFP^{-} Tcons in *Dgka$^{-/-}$z$^{f/f}$-Foxp3$^{YFPCre/YFPCre}$* and *Dgka$^{-/-}$z$^{f/f}$-Foxp3$^{YFPCre/+}$* mice but not in *Dgka$^{+/+}$z$^{f/f}$-Foxp3$^{YFPCre/YFPCre}$* mice. Ctrl: DNA from total LN cells (including both Tregs and Tcons) of *Dgka$^{-/-}$z$^{f/f}$-Foxp3$^{YFPCre/YFPCre}$* mice. MK: DNA ladder. S1 and S2 indicate different mice. One of two experiments of total four *Dgka$^{+/+}$z$^{f/f}$-Foxp3$^{YFPCre/YFPCre}$* mice and four αζDKO mice is shown. The Cre mediated recombination of floxed Dgkz allele was detected with two Dgkz primers (forward: 5′-GGCACACTGGAGACTCTCAC-3′ and reverse: 5′-CTAGCTTGGGCTGCAGGT-3′). The CD14 genomic DNA was amplified using the following primers: Cd14forward: 5′-GCTCAAACTTTCAGAATCTACCGAC-3′ and Cd14reverse: 5′-AGTCAGTTCGTGGAGGCCGGAAATC-3′. **B.** Variegated tdTomato expression in *Foxp3$^{YFPCre/YFPCre}$-Rosa26$^{LSL-tdTomato}$* mice**. C–F.** TCRβ$^{-/-}$ mice were coinjected with a mixture of CD45.1^{+}CD45.2^{-} WT CD4^{+} T cells, CD45.1^{-}CD45.2^{+} CD4^{+}YFP^{+} WT Tregs, and CD45.1^{+}CD45.2^{+} CD4^{+}YFP^{+} Treg-αζDKO Tregs and analyzed 8 weeks later. **C.** Representative FACS plots showing CD45.1 and CD45.2 staining in live gated mLN CD4^{+}TCRβ^{+} cells. **D.** Percentages of WT and αζDKO Treg-derived cells within adoptively transferred CD4^{+}TCRβ^{+} cells. **E.** FACS plots showing YFP levels in live gated cells derived from WT or αζDKO Tregs. **F.** Percentages of YFP^{-} exTregs. Data shown are representative or pooled from 5–7 experiments. *, $p<0.05$; **, $p<0.01$; ***, $p<0.001$ determined by two-tail unpaired Student t-test. **G, H.** 5X10^{5} Foxp3YFP^{+} Tregs from WT-*Foxp3$^{YFPCre/YFPCre}$* and *Dgka$^{-/-}$z$^{f/f}$-Foxp3$^{YFPCre/YFPCre}$* mice were separately *i.v.* injected into CD45.1^{+} C57BL6/J mice 4 hours after irradiation (400Rad). LNs from recipient mice were examined 14 days later. **G.** Representative FACS plots showing CD4^{+}TCRβ^{+} T cells. **H.** Scatter plots show mean ± SEM of data pooled from three experiments. *, $p<0.05$ determined by two-tail unpaired Student t-test.
